# Noninvasive KRAS mutation estimation in colorectal cancer using a deep learning method based on CT imaging

**DOI:** 10.1186/s12880-020-00457-4

**Published:** 2020-06-01

**Authors:** Kan He, Xiaoming Liu, Mingyang Li, Xueyan Li, Hualin Yang, Huimao Zhang

**Affiliations:** 1 The First Hospital of Jilin University, Department of Radiology, Changchun, China; 2 Jilin University, College of Electronic Science and Engineering, State Key Laboratory on Integrated Optoelectronics, No.2699 Qianjin Street, Changchun, China

**Keywords:** Colorectal Neoplasm, Mutation, Deep learning

## Abstract

**Background:**

The detection of Kirsten rat sarcoma viral oncogene homolog (KRAS) gene mutations in colorectal cancer (CRC) is key to the optimal design of individualized therapeutic strategies. The noninvasive prediction of the KRAS status in CRC is challenging. Deep learning (DL) in medical imaging has shown its high performance in diagnosis, classification, and prediction in recent years. In this paper, we investigated predictive performance by using a DL method with a residual neural network (ResNet) to estimate the KRAS mutation status in CRC patients based on pre-treatment contrast-enhanced CT imaging.

**Methods:**

We have collected a dataset consisting of 157 patients with pathology-confirmed CRC who were divided into a training cohort (n = 117) and a testing cohort (n = 40). We developed an ResNet model that used portal venous phase CT images to estimate KRAS mutations in the axial, coronal, and sagittal directions of the training cohort and evaluated the model in the testing cohort. Several groups of expended region of interest (ROI) patches were generated for the ResNet model, to explore whether tissues around the tumor can contribute to cancer assessment. We also explored a radiomics model with the random forest classifier (RFC) to predict KRAS mutations and compared it with the DL model.

**Results:**

The ResNet model in the axial direction achieved the higher area under the curve (AUC) value (0.90) in the testing cohort and peaked at 0.93 with an input of ’ROI and 20-pixel’ surrounding area. AUC of radiomics model in testing cohorts were 0.818. In comparison, the ResNet model showed better predictive ability.

**Conclusions:**

Our experiments reveal that the computerized assessment of the pre-treatment CT images of CRC patients using a DL model has the potential to precisely predict KRAS mutations. This new model has the potential to assist in noninvasive KRAS mutation estimation.

## Background

CRC is the third most commonly diagnosed malignancy and the fourth leading cause of cancer-related deaths in the world, and its burden is expected to increase by 60% to more than 2.2 million new cases and 1.1 million cancer deaths by 2030 [1]. The distinct heterogeneity of prognosis and treatment response has been observed in clinical practice among various CRC patients, even in those who share similar pathological stages and treatment modalities. KRAS is a small G protein that plays a role in the epidermal growth factor receptor (EGFR) pathway. KRAS mutations are acquired early during colorectal tumorigenesis. In approximately 40% of all metastatic colorectal cancer tumors, one of several heterozygous KRAS codon 12 or 13 mutations is detected [2]. Targeted treatments with anti-EGFR monoclonal antibodies (cetuximab and panitumumab) are recommended by the National Comprehensive Cancer Network (NCCN) for metastatic CRC patients whose tumors do not harbor KRAS mutations [3, 4]. Therefore, the detection of KRAS gene mutations in CRC is key to the optimal design of individualized therapeutic strategies.

Invasive colonoscopy or surgery for the biopsy is the limitation of KRAS mutational testing. The samples from these invasive procedures may be limited by intratumoral heterogeneity and may not sufficiently represent the exact macroscopic status of the entire tumor. Moreover, the NCCN [3] recommends that fresh biopsies should not be obtained solely for the purpose of KRAS genotyping. Histological biopsies for KRAS testing are usually performed before the initial treatment and cannot be repeated in the subsequent treatment. Thus, it is very useful to explore a noninvasive method to assist in the diagnosis of KRAS mutations. The noninvasive prediction of the KRAS status in CRC is challenging. Medical imaging technology provides some exploratory methods for the detection of the KRAS status. Several previous studies have used positron emission tomography (PET)/computed tomography (CT) with 18-F fluorodeoxyglucose PET/CT to assess KRAS mutation information in CRC [5–7]. An alternative CT-based radiomics approach for noninvasive KRAS mutation estimation in CRC has also been applied [8].

Artficial intelligence (AI) has become a hot topic in medical care support. Broadly speaking, machine learning can be divided into two major classes: radiomic analysis, which relies on multi-step pipelines, and the concept of the DL method, which simplifies this pipeline by learning predictive features on its own. Recent studies have shown the potential for DL in medical imaging due to its high performance in diagnosis, classification, and prediction [9–11]. Previous research has also shown the great potential of DL in the prediction of key molecular markers in gliomas [12, 13].A convolutional neural network (CNN) is a type of neural network developed specifically to learn hierarchical representations of imaging data. A ResNet is an effective exploration of a deep CNN, allowing the effective training of substantially deeper networks than those used previously while maintaining fast convergence times [14]. A ResNet has been used increasingly due to its utility and simplicity in clinical applications [15, 16].

To the best of our knowledge, there is no research on whether a CT-based DL model can predict the KRAS mutation status in CRC. Therefore, we developed and evaluated a ResNet model on contrast-enhanced CT before treatment to noninvasively predict the KRAS status in CRC. We also explored a radiomics model to compare with the DL model.

## Methods

### Data collection

This research was approved by the First Hospital of Jilin University medical ethics committee. All the patients involved in the study signed an informed consent. A total of 157 patients who had pathologically confirmed CRC and underwent KRAS mutation tests and contrast-enhanced CT pretreatment were identified retrospectively between December 2016 and January 2018. Inclusion criteria: (a) Archive data for patients with pathology-confirmed colorectal cancer from December 2016 to January 2018; (b) Patients before treatment who underwent a KRAS mutation test; (c) Pre-treatment contrast-enhanced CT available; and (d) Contrast-enhanced CT with a reconstruction slice thickness of 1.5 mm. Exclusion criteria: (a) CRC patients who underwent radiotherapy, chemotherapy or chemoradiotherapy before obtaining the pathological tissue sample; (b) Patients without enhanced CT before specimen collection. These patients were classified according to CT acquisition time and then assigned to either training cohort or testing cohort on a 3:1 ratio. There were 75 men and 42 women (mean age, 60 years; age range, 28 −88 years) assigned to the training cohort and 31 men and 9 women (mean age, 59 years; age range, 32 −73 years) assigned to the testing cohort. KRAS mutations (exons 2, 3, and 4) were analysed by a next-generation sequencing (NGS) method.

### CT technique

All patients underwent contrast-enhanced CT examinations using one of two 64-detector row spiral CT scanners (Philips, Brilliance iCT). CT scan was performed after 65s delay following intravenous injection of 100ml Iopromide (Uitravist-300; Bayer Schering Pharma, Berlin Germany) at a rate of 3ml/s for enhancement. The scanning parameters were as follows: 120 kV; 150 mAs; rotation time, 0.5 s; and matrix size, 512 ×512. The section thickness was 1.5 mm, and the interval was 3 mm.

### Data normalization

The portal venous phase images obtained from contrast-enhanced CT in the axial, coronal, and sagittal directions were first preprocessed by performing intensity normalization to reduce the noise and inconsistencies due to low-frequency non-uniformity or the inhomogeneity of intensities[17].The Z-score[18]method was applied to normalize the training and testing cohorts:

$$I'=\frac{I-\bar{I}}{\sigma_{I}} $$

where *I* indicates a slice from any cohort, $\bar {I}$ indicates the average greyscale value of the two cohorts and *σ*_1_ indicates the greyscale standard deviation of the two cohorts.

### ROI patch generation

Following normalization, the ROIs were manually delineated along the contour of tumor on the largest tumor cross section in axial direction, coronal direction and sagittal direction. Air area inside the tumor area was excluded from the contour. Two professional radiologists with 12 (reader 1, W.X.) and 7 (reader 2, X.X.Z.) years of experience delineated the ROI in a blinded fashion. The two-dimensional segmentation was completed using ITK-SNAP software (version 3.4; www.itk-snap.org). An independent samples t-test was used to evaluate the differences between the features generated by reader 1 and reader 2 (interobserver), and the differences between the twice-generated features by reader 1 (intraobserver). Inter- and intraclass correlation coefficients (ICCs) were used to assess the agreement of feature extraction. It was considered acceptable that the ICC is greater than 0.75.

To extract original patches, a minimum bounding rectangle was first drawn around each manual ROI. That ensured the entire tumor area, as well as the minimum peritumoral tissue, was captured. The region of the minimum bounding rectangle was cropped in this slice. Since the size of all the ROI patches was different, we counted the median height and median width from all the ROI patches for three groups. Then, nonlinear interpolation was used to resize the ROI patches for three groups. The ROI patch sizes after resizing were 60 ×60 pixels for the axial direction, 68 ×63 pixels for the sagittal direction and 72 ×67 pixels for the coronal direction.

**Fig. 1:**
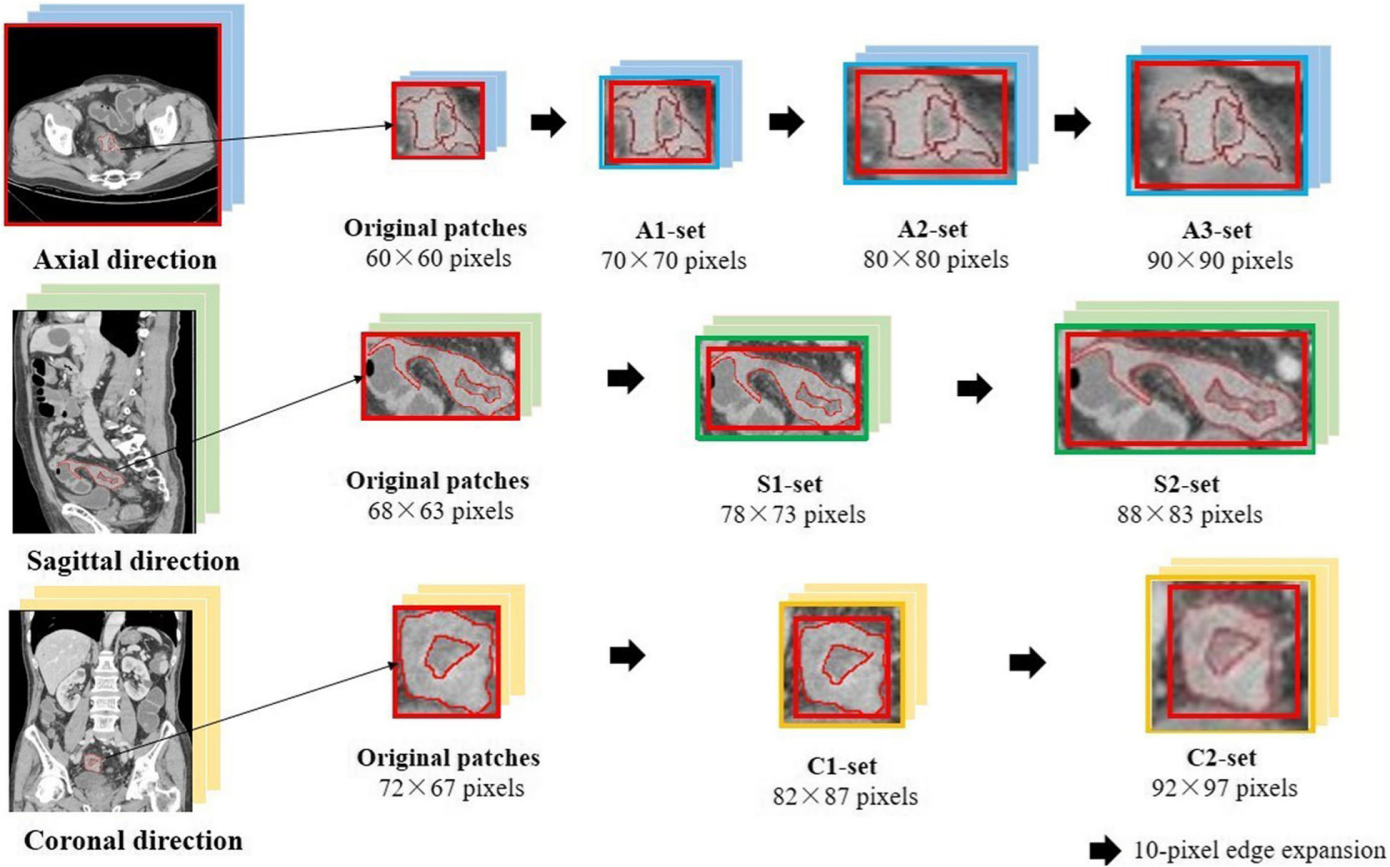
The flow chart of ROI-patch generation. A minimum circumscribed rectangle is established around each irregular ROI. Then, the boundary of the minimum rectangle is equally expanded with an interval of 10 pixels. This procedure is repeated for the three orthogonal directions (axial, sagittal, and coronal)

Several groups of expended ROI patches were generated for the ResNet model, to explore whether tissues around the tumor can contribute to cancer assessment. The minimum bounding rectangle was enlarged in both height and width, with an interval of 10 pixels. We cropped and resized the patches in the same manner as in the original group ROI patches in three directions. For axial images, we generated 3 groups of patches as the subsequent experimental dataset. The sizes were 70 ×70 pixels for A1-set, 80 ×80 pixels for A2-set and 90 ×90 pixels for A3-set. Similarly, we generated 2 groups of patches for sagittal images (78 ×73-pixel patches of S1-set and 88 ×83-pixel patches of S2-set) and coronal images (82 ×87-pixel patches of C1-set and 92 ×97-pixel patches of C2-set). The CT images of all patients in the training cohort were constructed using the above steps. The detailed workflow of ROI patch generation and relative expansion is shown in Fig. 1.

### Data augmentation

DL networks are often data-driven architectures, and data augmentation is an effective way to improve the model and reduce overfitting. Therefore, we augmented the training dataset by introducing random rotations and translations, generating ’new’ training data. The augmentation technique allows us to further increase the size of our training cohort. All images were converted into 5 pixels, 10 pixels, 15 pixels, and 20 pixels and rotated by 3 ^∘^, 6 ^∘^, 9 ^∘^, 12 ^∘^, and 15 ^∘^. Then, the above 9 transformations and original images were separately rotated by 1 ^∘^, 2 ^∘^, 3 ^∘^, 4 ^∘^, and 5 ^∘^. Therefore, the number of images in the primary cohort expanded to 50 times its initial scale.

### ResNet

**Fig. 2:**
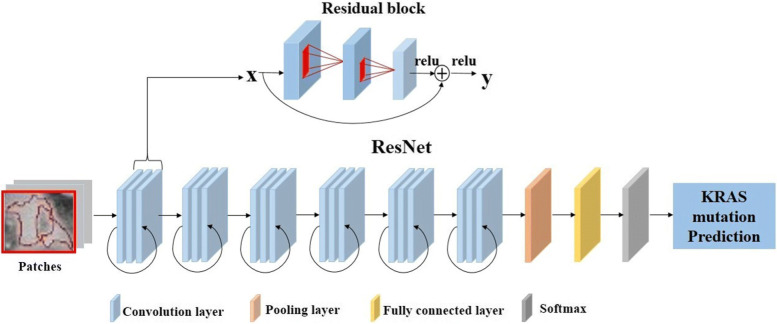
The structure of the employed residual neural network. There are six identity blocks, a pooling layer, a fully-connected layer and a softmax. Each identity block has three convolutional layers. The kernel size of all the convolution layers is 5 ×5. ReLu are adopted after every convolutional layer

A ResNet was applied to train the imaging data and build our neural network model. There are six residual learning blocks. As shown in Fig. 2, for input *x*, the residual block output *y* is defined as:

$$y=\sigma(F(x,W_{i})+x) $$

where the function is the *i*-th residual mapping and *σ* denotes the rectified linear unit (ReLU) [19] process. The kernel size of all the convolution layers is 5 ×5. Then,a max-pooling layer, a fully connected layer and a soft-max layer are implemented.

In the training phase, we trained the ResNet model with the constructed datasets in the portal venous phase images of the axial, coronal, and sagittal directions. The datasets in each direction contained both the original patch and the extended patch. We fed the ResNet with patches of different sizes and obtained 10 corresponding pre-trained models, which were models with original axial patches, A1-set, A2-set, and A3-set, original sagittal patches, S1-set and S2-set, and original coronal patches, C1-set and C2-set. In the testing phase, we evaluated the performances of the above 10 pre-trained models, respectively.

All experiments were performed on the workstation of a Windows 10 64-bit operating system with a 64-GB memory and an NVIDIA GeForce GTX 1080 GPU. Data normalization and ROI generation were performed in MATLAB 2016b. Data augmentation, training and testing for all the ResNet models were developed on the Keras library with a TensorFlow backend. When training the ResNet, the Adam optimization function was used with a batch size of 40 and a learning rate of 0.001.

### Radiomics model

We also explored a radiomics model with RFC to predict KRAS mutations and compared it with the DL model. Random forest classifier has been a prevalent data mining and statistical tool because of its transparency and great success in classification and regression task [20, 21]. A total of 1025 features, including tumor intensity, shape and size, texture, and wavelet characteristics, were extracted from the primary tumors based on the manually delineated ROI. Detailed descriptions of these features are shown in Supplementary Information 4.1. Feature selection and modelling were based on the training cohort. A univariate analysis was performed for each feature. Features with P values <0.05 were considered associated with KRAS mutations and were incorporated into the least absolute shrinkage and selection operator (LASSO) logistic regression model with 10-fold cross-validation. We established a radiomics model with an RFC according to the low-dimensional radiomics feature signature. The RFC consists of multiple classification and regression trees (CARTs), which are highly accurate and tolerant to exception values and noise without being prone to overfitting. Detailed descriptions of the radiomics method are shown in Additional file 1.

## Results

### Patient demographics

The demographic and tumor characteristics in the training and testing cohorts are listed in Table 1. Based on the results of KRAS status, the patients were classified into two groups: the mutated group and the wild-type group. Regarding gender, age and TNM stage, no demographic differences were observed between the two groups in both cohorts (p >0.05). However, for tumor size, statistical significance was found in both cohorts (P values <0.05). There were significant differences in tumor location between the two groups in the testing cohort, but these differences were not confirmed in the training cohort.

**Table 1:** Demographic differences in the training and testing cohorts No, number; m, median; SD standard deviation

Characteristics	Training cohort		*p* value	Testing cohort		*p* value
	Wild-type group	Mutated group		Wild-type group	Mutated group	
Gender (No [%])			*0.3239*			*0.4753*
Male	36(59.02)	38(67.86)		18(81.81)	13(72.22)	
Female	25(40.98)	18(32.14)		4(18.18)	5(27.78)	
Age (m ± SD)	59.80 ±11.05	60.33 ±9.84	*0.7853*	57.68 ±9.86	59.56 ±	*0.5389*
Tumor size, (cm ± SD)	3.49 ±1.21	3.05 ±1.18	0.0492*	3.55 ±1.29	4.8 ±1.74	0.0129*
Tumor location (No [%])			*0.8668*			0.0419*
Ascending colon	4(6.56)	5(8.93)		0(0.00)	4(22.22)	
Transverse colon	3(4.92)	3(5.36)		1(4.55)	0(0)	
Descending colon	4(6.56)	2(3.57)		4(18.18)	3(16.67)	
Sigmoid colon	27(44.26)	25(44.64)		11(50.00)	4(22.22)	
Rectum	15(24.60)	18(32.14)		6(27.27)	4(22.22)	
Cecum	7(11.48)	4(7.14)		0(0.00)	3(16.67)	
T category (No [%])			*0.0928*			*0.2645*
T1	1(1.64)	0(0.00)		0(0.00)	0(0.00)	
T2	5(8.20)	2(3.57)		2(9.09)	0(0.00)	
T3	41(67.21)	49(87.5)		17(77.27)	13(72.22)	
T4	13(21.31)	5(8.93)		3(13.63)	5(27.78)	
N category (No [%])			*0.2361*			*0.3657*
N0	19(31.15)	12(21.43)		1(4.55)	0(0)	
N1, N2	42(68.85)	44(78.58)		21(95.45)	18(100)	
M category (No [%])			*0.6719*			*0.7814*
M0	38(62.30)	37(66.07)		15(68.18)	13(72.22)	
M1	23(37.70)	19(33.93)		7(31.82)	5(27.78)	

*p* value <0.05 indicates a significant difference in patients’characteristics between the primary cohort and testing cohort. ∗, *P*< 0.05.

### Predictive performance of the ResNet classification model

**Fig. 3:**
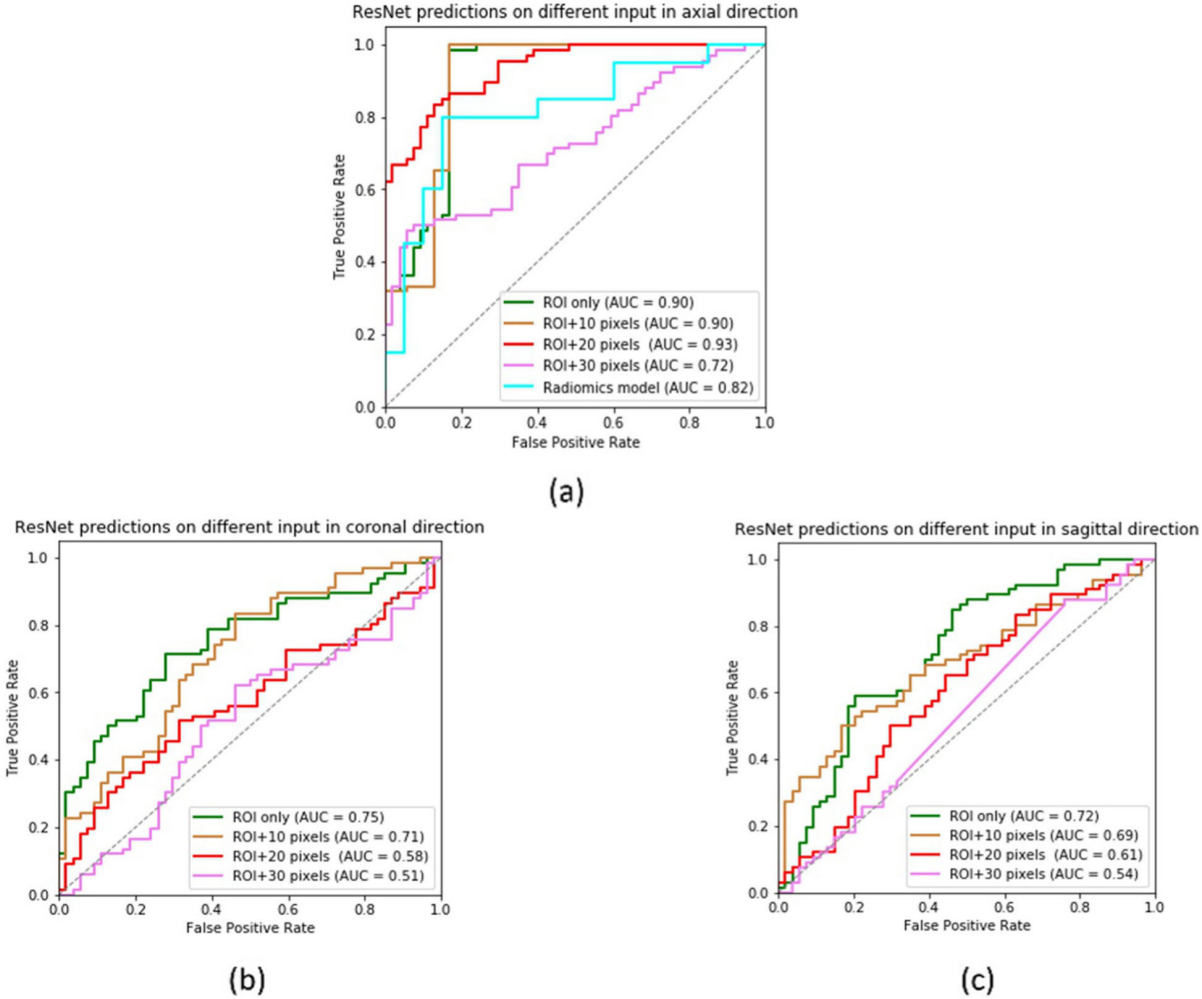
ROC curves for four KRAS mutations predicted by the residual neural network and radiomics models in testing cohort. **a** ResNet and raidiomics predictions on different input in axial direction. **b** ResNet predictions on different input in coronal direction. **c** ResNet predictions on different input in sagittal direction

**Fig. 4:**
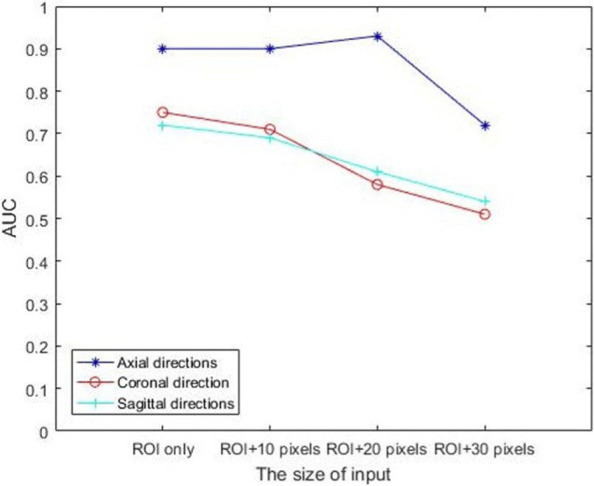
The line chart of AUC values for each CNN with different inputs in testing cohort. ResNet model in the axial direction reached the higher AUC value compared with the coronal and sagittal

We investigated the predictive model for KRAS gene mutation for CRC patients based on CT images in three positions: axial direction, coronal direction and sagittal direction. Metrics of the AUC, sensitivity and specificity were used to evaluate the performances of the networks. In addition, several groups with different input sizes were carried out to explore the effect of the surrounding tissue on classification accuracy. All the results for the testing cohort are displayed in Table 2. Figure 3a shows the AUC in the axial direction peaked at 0.93 when the input was ’ROI and 20-pixel’, and the increase in the AUC value was limited by the expansion of the input size. Figure 3b and c show that the inclusion of surrounding tissues did not contribute to the ResNet model either in the coronal or sagittal direction. Figure 4 shows that the ResNet model in the axial direction reached the higher AUC value compared with the coronal and sagittal positions.

**Table 2:** Performance of Models of the KRAS mutation prediction in testing cohorts

Model		AUC	Sensitivity	Specificity
Radiomics model		0.82	0.7	0.85
ResNet model				
Axial directions	ROI only	0.9	0.65	0.83
	ROI + 10 pixels	0.9	0.67	0.83
	ROI + 20 pixels	0.93	0.59	1
	ROI + 30 pixels	0.72	0.67	0.63
Coronal direction	ROI only	0.75	0.79	0.56
	ROI + 10 pixels	0.71	0.83	0.46
	ROI + 20 pixels	0.58	0.45	0.7
	ROI + 30 pixels	0.51	0.7	0.28
Sagittal directions	ROI only	0.72	0.56	0.8
	ROI + 10 pixels	0.69	0.61	0.65
	ROI + 20 pixels	0.61	0.58	0.57
	ROI + 30 pixels	0.54	0.89	0.13

### Predictive performance of the radiomics model

For the feature selection of the high-dimensional radiomic features of the training cohort, 6-dimensional features of P <0.05 are obtained by t-test. Then, the 4-dimensional radiomics feature signatures are selected by LASSO regularization with 10-fold cross-validation. The feature names, values and coefficients of the radiomics feature signatures are shown in Additional file 1. We use RFC as the basic model. The model parameters are n_estimators = 200, max_depth = 4; that is, the number of CART is 200, and the maximum tree depth is 4. In the training cohort, the AUC was 0.945 (sensitivity: 0.75; specificity: 0.94), and in the testing cohort, the AUC was0.818 (sensitivity: 0.70; specificity: 0.85).In comparison, the ResNet model showed better predictive ability. The ROC curves are shown in Fig. 3a.

## Discussion

Targeted treatments with anti-EGFR monoclonal antibodies (cetuximab and panitumumab) are recommended by the NCCN for metastatic CRC patients whose tumors do not harbour KRAS mutations [3, 4].The determination of the KRAS mutational status in CRC patients is essential for the management of CRC. However, several issues with KRAS testing limit its utility, as described previously. The aim of our study was to employ a ResNet model to provide a noninvasive preoperative prediction of the KRAS mutational status. To the best of our knowledge, this is the first study concerning the predictive value of DL based on CT images in CRC. Previous studies have attempted to investigate the relationship between image characteristics and genetic mutations [6, 22, 23] or pathological subtype [24, 25]. Compared with other medical imaging technology, one of the challenges of DL is the requirement of a large training dataset. A limited number of suitable patients might lead to insufficient training data. Therefore, in the study, data augmentation was used to increase the size of the training dataset and prevent overfitting. Besides, the ResNet with simple architecture and short time consumption was enough to learn the predictive features according to the satisfactory results in our study and the other researchers [26, 27].

To explore the lesion-based prediction in three positions: axial, coronal, and sagittal, and the influence of surrounding tissues on the classified results, we cropped several groups of patches around the manually annotated ROI. When the input was ‘ROI only’, the ResNet model in the axial direction reached the higher AUC value compared with the coronal and sagittal direction. That shows the reconstructed images based on 2D axial images do not contribute to the improvement of model performance. One interesting finding is that the inclusion of surrounding tissues contributed to the ResNet just in the axial direction, and the continuous expansion of the input size limited the improvement of AUC.The AUC in the axial direction peaked at 0.93 when the input was ‘ROI and 20-pixel’, which indicated that the surrounding tissue could contribute to the model’s performance. However, when further expand the surrounding edge information, more noise may be introduced, so the classification ability of the network decreased. The inclusion of surrounding tissues for KRAS mutational classification in CRC which has biological importance should be explored further.

Our study also compared the performances of the DL method and the CT-based radiomics method. Some studies found that slice thickness affected radiomic feature values and the performance of diagnostic models [28, 29]. To reduce the effects of reconstruction slice thickness on the performance of the radiomic model, we only included images of the same thickness. However, the diagnostic performance of the ResNet model with 2D input data was unaffected by slice thickness. Thus, the proposed deep learning model is suitable for routine CT imaging with other slice thickness. In our study, the proposed CT-based radiomics signature incorporated four radiomics features: two high-dimensional features obtained by the LoG and two high-dimensional features obtained by the wavelet transform, which are all conducive to classification, shown in Additional file 1. We chose the RFC as the machine learning classifier. The RFC consists of multiple CARTs, and each CART trains the sub-classifier by bootstrapping. Finally, the RFC predicts results through the voting of each CART, so it has good generalization [30]. In the training cohort, the AUC was 0.945 (sensitivity: 0.75; specificity: 0.94), and in the testing cohort, the AUC was 0.818 (sensitivity: 0.70; specificity: 0.85). These results are consistent with the results of other investigators [8]. The clinical background and tumor stage were not used in the radiomics model or the ResNet model to ensure the consistency of the input data type. The comparison of the two models indicates that the CT-based DL model could reach the predictive level of the radiomics model in predicting the KRAS mutation status of CRC patients, while the DL can simplify the multi-step pipeline of the conventional radiomics method with little pre-processing and relatively greater reproducibility [31].

There are several limitations to this preliminary study. First, the number of study subjects was limited, and although data augmentation was performed to increase the size of the training cohort, it was still difficult to observe a robust outcome. However, the deep neural network has achieved good performance in many clinical medical applications, both with large and relatively small sample sizes [10, 32, 33]. Second, the input data type was 2D and not 3D in the DL model. 3D deep learning requires significantly higher computation power than sequential 2D image analyses. A further study with the differences between 2D and 3D models of DL in predicting KRAS mutations in CRC will need to be undertaken. Third, our study analyzed the KRAS gene status of only the RAS-RAF-MAPK pathway. The V-raf murine sarcoma viral oncogene homolog B (BRAF) mutation is another marker of anti-EGFR resistance in non-first-line treatment of metastatic CRC. We did not accumulate enough BRAF mutation cases because of its low prevalence in CRC. Fourth, we included only a single-center cohort with an internal testing set. In the future, large, multi-center cohorts should be recruited for evaluation.

## Conclusions

The aim of the present research was to develop and evaluate a DL model on contrast-enhanced CT before treatment to noninvasively predict the KRAS status in CRC. We also explored a radiomics model with RFC to compare with the DL model (ResNet). The notable findings of our study can be summarized as follows:

(1) Computerized assessment of the pre-treatment CT images of CRC patients using DL has the potential to precisely predict KRAS mutations. (2) The 2D ResNet model in the axial direction reached the higher AUC value compared with the coronal and sagittal direction. (3) The continuous expansion of the input size with surrounding tissues limited the performance of the ResNet model. (4) CT-based DP model may reach the predictive level of the radiomics model in predicting the KRAS mutation status of CRC patients, while the DL can simplify the multi-step pipeline of the conventional radiomics method.However, there are still several limitations in the DL research field, including the insufficient number of patients, and further studies should be performed to optimize and verify its utility.

## Supplementary information

**Additional file 1** Supplementary file.

## Data Availability

The datasets generated and/or analysed during the study are not publicly available due to patient privacy protection, but are available from the corresponding author on reasonable request.

## Abbreviations

RFC: Random forest classifier
AUC: Area under the curve
EGFR: Epidermal growth factor receptor
NCCN: National comprehensive cancer network
PET: Positron emission tomography
CT: Computed tomography
AI: Artificial intelligence
CNN: Convolutional neural network
LASSO: Least absolute shrinkage and selection operator
CARTs: Classification and regression trees
BRAF: V-raf murine sarcoma viral oncogene homolog B
