# Supplementary material for: Noninvasive KRAS mutation estimation in colorectal cancer using a deep learning method based on CT imaging
Source: BMC Med Imaging. 2020 Jun 1;20:59. doi: 10.1186/s12880-020-00457-4 (PMC7268438; doi:10.1186/s12880-020-00457-4)
Supplement: Supplementary file 1 — Additional file 1 Supplementary file. [file 12880_2020_457_MOESM1_ESM.docx]

**Additional file 1 ：**

1. Feature extraction, feature selection, and radiomics model building

1.1 Radiomics feature extraction

1.2 Data standardization and feature selection

1.3 Model establishment and performance evaluation

2. Supplementary figures and tables

2.1. Table S1. Feature names, values and coefficients of radiomics feature signature

2.2 . Figure S1. Feature selection using the LASSO regression method

2.3 . Figure S2. ROC curves of the radiomics model

3. References

**1. Feature extraction, feature selection, and radiomics model building**

1.1 Radiomics feature extraction

Based on the extracted ROI and original CT images, we extracted 1025 -dimensional radiomic features from a single Digital Imaging and Communications in Medicine (DICOM) image of each patient after the conversion of the dataset by using the PyRadiomics package in Python 3.6 software, including the following:

1. Wavelet transform
2. Laplacian of Gaussian (LoG)
3. First-order texture features: mean, entropy, skewness, etc.
4. Shape features: surface area, sphericity, flatness, etc.
5. Grey level co-occurrence matrix: joint average, difference entropy, correlation, etc.
6. Grey level size zone matrix: grey level non-uniformity, zone entropy, grey level variance, etc.
7. Grey level run length matrix: grey level non-uniformity, run variance, short run emphasis, etc.

1.2 Data standardization and feature selection

Because the absolute values of the original radiomic features were quite different, the original data was standardized before data processing. We first standardized the training set (n=116) and then normalized the test set (n=40) with the mean and variance obtained from the training set. The standardization process is shown in formula (1).

$x^{*}=\frac{x-\mu}{\sigma}$ (1)

The x* represents the normalized data, x represents the raw data, μ represents the mean, and σ represents the standard deviation.

Since too many redundant features will cause a large interference in the final model, the dimension reduction of high-dimensional features is required before modelling. We reduced the dimension of the training set separately to ensure that the test set does not participate in the model establishment. First, the 1025-dimensional features were pre-selected in the training set; that is, independent t-tests(1) were performed on every original feature first, resulting in significantly correlated features with P<0.05. Then, the features obtained by pre-selection were selected by LASSO(2, 3) with 10-fold cross-validation. By the mean curve of 10-fold cross-validation, the alpha value of the minimum mean square error was found, and then the low-dimensional radiomics feature signature and feature coefficients were determined. The LASSO dimension reduction is shown in equation (2); that is, the regularized L1 norm is introduced under the condition that the mean square error is guaranteed to be small, and the sparse solution is more easily obtained.

$\min_{w}\sum_{i=1}^{m} \left( y_{i}-w^{T}x_{i} \right)^{2}+\alpha\left\| \omega\right\|_{1}$ (2)

1.3 Model establishment and performance evaluation

We built models according to low-dimensional radiomics feature signatures that are based on the RFC(4). The RFC consists of multiple CARTs, and each CART trains the sub-classifier by bootstrapping. The calculation process of the RFC model is described in the literature(5, 6). We used RFC as the basic model, with the model parameters set to n_estimators = 200, max_depth = 4; that is, the number of CART is 200, and the maximum tree depth is 4. Finally, the RFC predicts results through the voting of each CART, so it has good generalization. We used the ROC curve and AUC as indicators to evaluate model performance.

**2. Supplementary figures**

| Feature name | coefficient |
| --- | --- |
| log-sigma-1.0-mm-3D_glszm_GrayLevelNonUniformity | -0.04007475 |
| log-sigma-3.0-mm-3D_glszm_GrayLevelNonUniformity | -0.03885293 |
| wavelet-LLL_glrlm_GrayLevelNonUniformityNormalized | 0.0448999 |
| wavelet-LLH_firstorder_InterquartileRange | -0.0509126 |

Table S1. Feature names, values and coefficients of radiomics feature signature


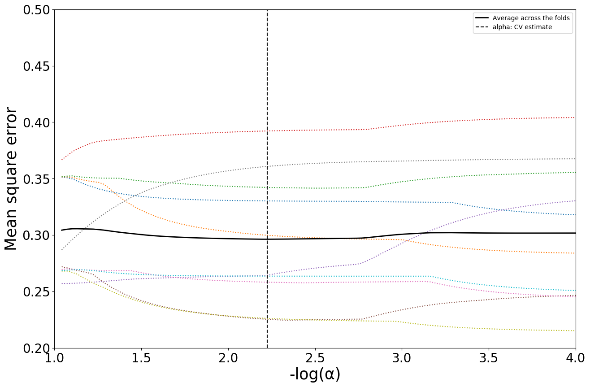

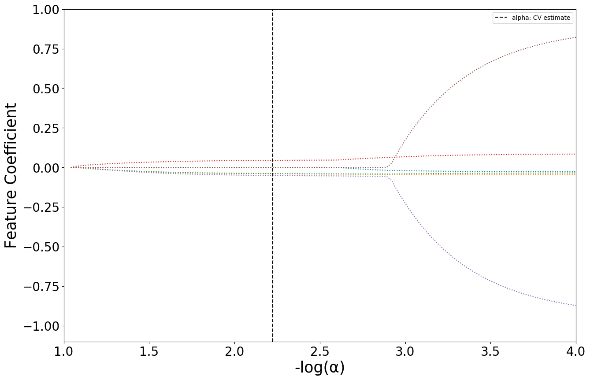


Figure S1. Feature selection using the LASSO regression method. We used 10- fold cross validation in the LASSO model for the selection of the conditioning parameters (Lambda). The AUC was plotted versus log(Lambda) by using the minimum standard and the minimum standard of 1 standard error (1 - SE standard) to draw the vertical line with the best value. A Lambda value of 0.00597, with log(Lambda) -2.22403 was chosen (1 - SE standard) according to the 10-fold cross validation


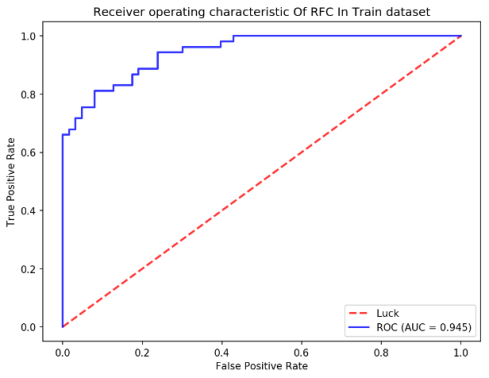


(a)


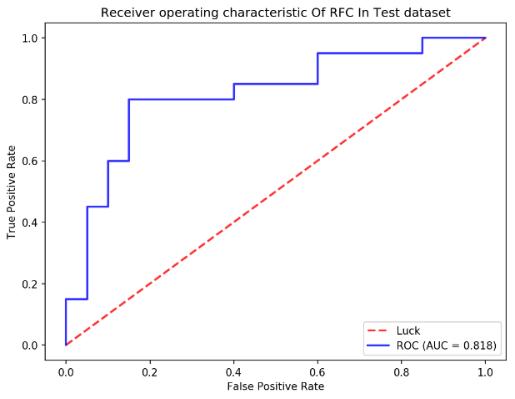


(b)

Figure S2. ROC curves of the radiomics model in the training (A) and validation (B) cohorts. The AUC of the training cohort was 0.94 and that of the validation cohort was 0.82.

**3.References**

1. Boonstra K, Weersma RK, van Erpecum KJ, Rauws EA, Spanier BWM, Poen AC, et al. Population-Based Epidemiology, Malignancy Risk, and Outcome of Primary Sclerosing Cholangitis. Hepatology. 2013;58(6):2045-55.

2. Ma C, Huang J. Asymptotic properties of Lasso in high-dimensional partially linear models. Science China-Mathematics. 2016;59(4):769-88.

3. Harrell FE. Regression Modeling Strategies: With Applications to Linear Models, Logistic and Ordinal Regression, and Survival Analysis, 2nd Edition. Regression Modeling Strategies: With Applications to Linear Models, Logistic and Ordinal Regression, and Survival Analysis, 2nd Edition. Springer Series in Statistics2015.

4. Fang K, Wu J, Zhu J, Xie B, editors. A review of technologies on random forests. Statistics & Information Forum; 2011.

5. Breiman L. Random forests. Machine learning. 2001;45(1):5-32.

6. Liaw A, Wiener M. Classification and regression by randomForest. R news. 2002;2(3):18-22.
